# Eigen-guided transformer: A data-driven approach for chronic kidney disease forecasting

**DOI:** 10.1371/journal.pone.0348886

**Published:** 2026-05-21

**Authors:** Fahman Saeed, Sultan Aldera

**Affiliations:** Computer Science Department, College of Computer and Information Sciences, Imam Mohammad Ibn Saud Islamic University (IMSIU), Riyadh, Saudi Arabia; Polytechnic University of Marche: Universita Politecnica delle Marche, ITALY

## Abstract

Accurate prediction of Chronic Kidney Disease (CKD) development is essential for prompt therapeutic intervention; nevertheless, it is difficult due to the highly individualized disease trajectories and intricate multivariate risk profiles. Contemporary deep learning models provide substantial predictive capability; yet, they frequently exhibit computational inefficiency and a lack of interpretability, akin to a “black box.” To overcome these limitations, we present the Eigen-Guided Transformer, an innovative architecture that systematically enhances model topology by data-driven eigenvalue analysis of clinical feature correlations. Our methodology independently establishes: (1) the optimal quantity of attention heads by ascertaining the intrinsic dimensionality necessary to account for 95% of data variance (h = 11), (2) an eigenvector-informed weight initialization to encapsulate a priori feature interactions, and (3) validation-driven depth optimization. Upon assessing daily-aggregated longitudinal sequences from the MIMIC-IV dataset for creatinine prediction, the model attained a Mean Squared Error (MSE) of 0.089 ± 0.004 and a Mean Absolute Error (MAE) of 0.132 + 0.0001, indicating an 11.1% enhancement over the most robust baseline (TFT). The Eigen-Guided Transformer exhibited exceptional parameter efficiency, achieving a FLOPs/accuracy ratio of 142.4M, markedly lower than that of TFT (300M) and Autoformer (1.0B). External validation of the eICU dataset demonstrated strong cross-institutional generalizability (MSE 0.0117 ± 0.0001; MAE 0.0254 + 0.0008). To guarantee clinical dependability, we incorporated Monte Carlo Dropout for uncertainty quantification, resulting in precisely calibrated prediction intervals with 95.8% observed coverage. Additionally, a demographic fairness audit encompassing age, gender, and ethnicity demonstrated equitable performance, with a maximum discrepancy ratio of merely 1.32. The Eigen-Guided Transformer offers a transparent, efficient, and reliable paradigm for the individualized therapy of chronic renal disease by matching attention mechanisms with the intrinsic biological structure of clinical data.

## 1 Introduction

Chronic Kidney Disease (CKD) denotes a chronic decline in renal function, marked by the gradual impairment of the kidneys’ capacity to filter waste products from the bloodstream. The disease advances through five stages, ranging from mild kidney impairment (Stage 1) to total kidney failure necessitating dialysis or transplantation (Stage 5, known as end-stage renal disease or ESRD). A significant problem in chronic kidney disease therapy is the considerable variability in progression rates among patients: some maintain stability for years with negligible decrease, while others advance swiftly to end-stage renal disease within months. The variability necessitates precise, individualized forecasting of illness progression for prompt clinical intervention, treatment strategy, and resource distribution [1].

Contemporary clinical practice predominantly depends on the periodic assessment of renal biomarkers, specifically serum creatinine and estimated glomerular filtration rate (eGFR), to monitor disease advancement. These data, however, reflect kidney function at specific timepoints and do not directly forecast future deterioration. Clinicians require predictive models that may foresee when a patient is prone to swift deterioration, facilitating preventive measures such as drug alterations, dietary changes, preparation for renal replacement treatment, or transplant assessment [2]. Predictive tools must reconcile accuracy with interpretability and computational feasibility to be viable in real-world healthcare environments.

The prevalence of chronic kidney disease (CKD) is significant and increasing globally. Presently, about 1.6 million persons undergo renal replacement treatment (RRT), primarily through hemodialysis, with 90% residing in affluent countries. In 2021 [2], chronic kidney disease diagnoses resulted in 1.5 million deaths, with forecasts suggesting an escalation to 2.2 million in the most optimistic scenario and 4 million in the most pessimistic scenario by 2040. The prevalence is particularly concerning in areas like the Middle East and North Africa, where rates exceed global averages due to increased instances of diabetes, hypertension, and obesity. Over 1.6 million patients worldwide receive hemodialysis for end-stage renal illness. 90% of patients live in high-income countries, and 56% are in only five (the U.S., Japan, Brazil, Italy, and Germany), which make up only 12% of the global population [1].

Due to the clinical necessity of precise CKD progression prediction and the expanding disease burden, researchers have developed computational methods to predict renal function decline. Tangri et al. (2011) [3] formulated the Kidney Failure likelihood Equation (KFRE), which employs demographic and clinical data to forecast the likelihood of kidney failure in individuals with chronic kidney disease stages 3–5. This model has been verified in several populations and continues to be a clinical standard, but its c-statistic often varies from 0.85 to 0.90, suggesting potential for enhance.

Precise prediction of CKD progression is essential for clinical decision-making and individualized treatment strategies. The development of the disease from early stages to end-stage renal disease varies considerably among people, rendering prediction especially difficult. Conventional clinical prediction models, such as the Kidney Failure Risk Equation (KFRE), have enhanced predictive accuracy. In a cohort of 603 children (62.7% male, median age 12 years) with a median eGFR of 44 mL/min/1.73 m², 75.8% exhibited a nonglomerular etiology of chronic kidney disease (CKD), 23.9% progressed to end-stage renal disease (ESRD) after 5 years, and the 4-variable Kidney Failure Risk Equation (KFRE) effectively predicted ESRD risk (C-statistics: 0.90, 0.86, 0.81 for 1-, 2-, and 5-year assessments) [4]. Ravizza et al. (2019) [5] utilized random forests and gradient boosting machines on electronic health record data, attaining an AUC of 0.90 for forecasting CKD progression. Xiao et al. (2019) employed support vector machines on a dataset comprising 551 patients, attaining an accuracy of 91.7% in diagnosing phases of chronic kidney disease (CKD).

Recent advancements in machine learning have demonstrated potential for anticipating chronic kidney disease (CKD). Deep learning methodologies, especially transformer-based architectures, have exhibited exceptional efficacy in identifying intricate temporal patterns within clinical data. Choi et al. (2024) [6] introduced a transformer architecture for disease progression modeling that surpassed conventional recurrent neural networks by adeptly mimicking irregular clinical visits. Specialized architectures such as Autoformer have been created for time-series forecasting, utilizing decomposition techniques to address seasonal patterns and long-term trends in clinical data.

Although conventional machine learning techniques have enhanced statistical benchmarks, they continue to face challenges in accurately modeling the intricate temporal correlations seen in longitudinal clinical data. This constraint has prompted the investigation of deep learning architectures. Norouzi et al. (2019) [5] created a recurrent neural network model that attained an AUC of 0.92 for forecasting CKD progression based on sequential laboratory measures. Makino et al. (2019) [7] utilized a deep convolutional neural network on longitudinal data from 9,497 patients, exhibiting enhanced performance (AUC 0.93) relative to conventional statistical methods (AUC 0.89).

Transformer models have emerged as particularly interesting architectures for time-series forecasting inside deep learning. Initially shown by Vaswani et al. (2017) [8] for natural language processing applications, have recently been successfully adapted for time series forecasting. The self-attention mechanism in transformers facilitates the capture of long-range relationships in sequential data, circumventing the constraints of recurrent structures. Luo et al. (2018) [9] created a transformer-based model for mortality prediction utilizing ICU time series data, attaining an AUC of 0.85, in contrast to 0.82 for LSTM models. Transformer by Li et al., 2019 [10] modified the original transformer architecture for multivariate time series forecasting, demonstrating enhanced performance compared to LSTM and CNN-based models.

Numerous specialized transformer variations have been created explicitly for time series forecasting: Informer by Zhou et al., 2021 [11] presented a ProbSparse self-attention method to enhance efficiency in long sequence forecasting, decreasing the temporal complexity from O(L²) to O(L log L), where L denotes the sequence length. Autoformer by Wu et al., 2021 [12] integrated a decomposition architecture with auto-correlation methods for seasonal time series, attaining state-of-the-art performance across many benchmarks. Liu et al., 2022 [13] introduced a Pyraformer, which is a pyramid attention module to capture multi-scale temporal dependencies, exhibiting enhanced performance in both short-term and long-term forecasting tasks. The Time-Series Choi et al. (2020) [14] utilized transformers on electronic health data for illness progression modeling in the healthcare sector, surpassing conventional RNNs by effectively catching intricate temporal patterns in irregular clinical visits. TFT {Lim, 2021 #322} [15]  is parameter-efficient (100K-2M parameters) yet computationally intensive (O(n²) complexity), rendering it appropriate for medical applications when interpretability warrants the computational

Neural Architecture Search (NAS) has arisen as a potential method for the automated construction of neural network topologies. Nonetheless, the majority of NAS approaches are computationally demanding and fail to properly account for the statistical characteristics of the data [16].

Numerous works have investigated data-driven methodologies for neural architecture design: DARTS (Liu et al., 2019) [17] introduced a differentiable architectural search technique that markedly decreased the computational expense of NAS, however still necessitated considerable GPU resources. Neural Architecture Optimization (Luo et al., 2018) [18] presented a gradient-based NAS method employing an encoder-decoder framework to enhance the efficiency of architecture space exploration. Mellor et al. (2021) [19] introduced a method for predicting neural network performance without training by examining the correlation pattern of activations, termed Correlation-Based Architecture Selection. Dataset2Vec (Jomaa et al., 2019) [20] introduced a meta-learning methodology that correlates dataset attributes with optimum neural architectures.

Numerous research have investigated the application of eigenvalue analysis in neural network construction, which is pertinent to our work. Spectral-Pruning (Wang et al., 2019) [21] employed eigenvalue decomposition to detect and eliminate redundant neurons in neural networks, attaining a parameter reduction of up to 90% with negligible performance degradation. The Eigenvalue-Based Architecture Design (Zhang et al., 2020) [22] suggested utilizing the eigenspectrum of the feature covariance matrix to ascertain the width of neural network layers. The study “Spectral Analysis for Attention Mechanisms.

Predicting subsequent creatinine levels in the ICU encompasses both acute kidney injury dynamics and chronic renal disease processes. Although this does not equate to modeling long-term outpatient CKD progression, precise renal biomarker prediction in ICU patients with pre-existing CKD or AKI risk is clinically significant—it facilitates early detection of deterioration, directs fluid and medication management, and advises on the timing of nephrologist consultations. Expanding this methodology to outpatient longitudinal chronic kidney disease populations becomes a significant future objective.

Our research advances existing methodologies by utilizing eigenvalue [23,24] decomposition specifically for the design of transformer architecture in the realm of CKD forecasting. In contrast to earlier research that concentrated on pruning current designs or defining layer widths, we employ eigenvalue analysis to ascertain the ideal quantity of attention heads and initialize attention weights, yielding a more efficient and effective model for clinical time series data.

Despite these technological advancements, contemporary transformer-based methodologies for chronic kidney disease forecasting are impeded by various intrinsic limits that obstruct their clinical implementation and predicted precision. Recent research on EHR-based forecasting for CKD and other chronic diseases increasingly utilizes regularly sampled, longitudinal time-series data with imputation techniques such as forward-filling and interpolation to create comprehensive patient histories for modeling [25–27]. Nevertheless, a restricted number of papers offer entirely repeatable pipelines featuring daily aggregation and ongoing regression targets, highlighting the originality and rigor of our suggested methodology. Conversely, generic architecture design: Most transformer models have static configurations that do not accommodate the distinctive characteristics of CKD data. The number of attention heads, embedding dimensions, and transformer blocks is often determined through empirical trial rather than data-driven approaches. Inefficient Parameter Utilization: Conventional transformers possess millions of parameters, many of which may be redundant for chronic renal disease prediction. This leads to increased computational requirements and the potential for overfitting, especially when clinical data is limited. Limited Interpretability: The obscure nature of many deep learning models hinders their application in healthcare settings where transparency is crucial for decision-making. Suboptimal performance on clinical time series: generic transformer architectures designed for natural language processing or conventional time series may inadequately capture the unique characteristics of clinical data, such as irregular sampling and complex feature interactions.

To mitigate these limitations—such as static designs, ineffective parameter use, and inadequate performance on clinical time series—we present the Eigen-Guided Transformer. This approach tailors attention heads and weights by eigenvalue decomposition of the data’s correlation structure, guaranteeing efficient representation and enhanced forecasting precision. This section outlines our process for developing and training the model. Our principal contributions are:

(1) methodical customization of transformer heads and weights through eigenvalue analysis.
(2) architectural optimization utilizing validation-based search with early termination.
(3) an innovative data pipeline that incorporates daily aggregation and imputation of patient records, facilitating continuous regression targets for forthcoming biomarker values.
(4) empirical evidence indicates that our methodology outperforms existing models (ARIMA, LSTM, Autoformer) in forecasting CKD progression.
(5) Uncertainty quantification through MC Dropout with calibration analysis.
(6) External validation on the eICU dataset.
(7) Subgroup fairness analysis across demographic groups
(8) Sensitivity analysis validating the eigenvalue-guided head selection.

This approach has potential applications beyond CKD to other chronic diseases where accurate forecasting is essential for clinical decision-making and personalized treatment planning.

The remainder of this paper is organized as follows: Sect 1 introduces the topic; Sect 2 delineates the recommended methodology; Sect 3 shows the results of the experiments and engages in discussion; Sect 4 conclude the research.

## 2 Methodology

In light of the challenges involved in precisely forecasting CKD progression from longitudinal EHR data, we establish a systematic, data-driven approach that utilizes the statistical characteristics of the data to inform the design of Transformer architecture. Our methodology as presented in Fig 2 and Algorithm 1 encompasses dataset preparation, eigenvalue-directed customization, and validation-driven depth search, as detailed below.

**Fig 1:**
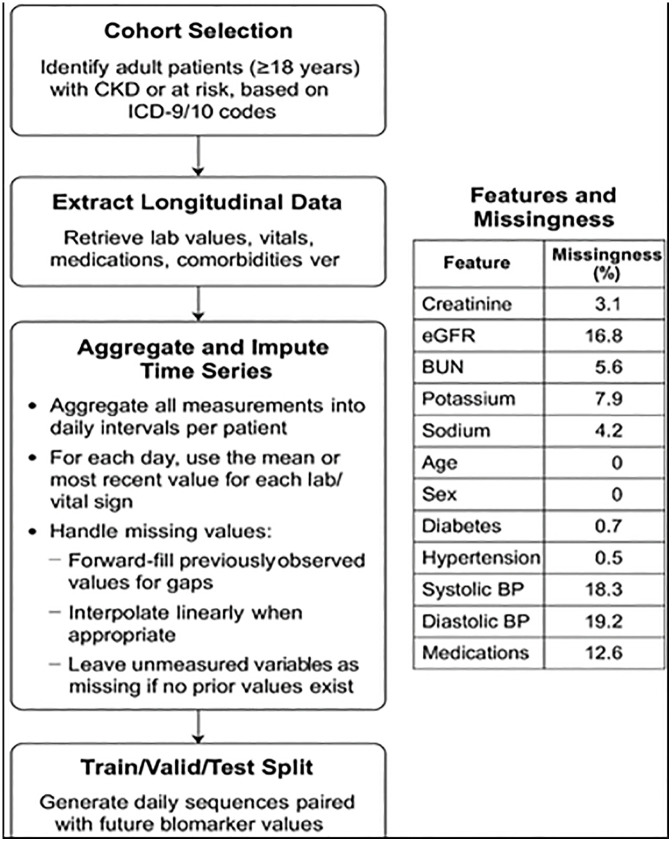
Pipeline for dataset preparation for chronic kidney disease forecasting utilizing MIMIC-IV electronic health records. The workflow delineates: (1) patient identification via ICD-9/10 diagnosis codes, (2) extraction of time-stamped laboratory measurements (creatinine, eGFR, potassium, BUN), (3) daily aggregation of all measurements per patient, (4) three-tiered missing value management (forward-filling, linear interpolation, and selective deletion), and (5) formulation of longitudinal sequences with continuous regression targets for prospective biomarker prediction.

### 2.1 Dataset description

To prepare the MIMIC-IV dataset for forecasting the progression of chronic kidney disease (CKD) as a continuous outcome and the eICU Collaborative Research Database (v2.0) for external validation. Current CKD prediction methods have limitations. Recently, EHR-based forecasting for CKD and other chronic diseases uses regularly sampled, longitudinal time-series data with imputation methods like forward-filling and interpolation to construct detailed patient histories for modeling [25–28]. We initially identified adult patients (≥18 years) with CKD or those at risk, utilizing ICD-9/10 diagnosis codes from the diagnoses_icd table and cross-referencing demographic data from the patients table. For each chosen patient, we retrieved all time-stamped laboratory measurements pertinent to renal function (including creatinine, eGFR, potassium, and blood urea nitrogen) from the labevents table and associated them with the respective hospital admissions and ICU stays utilizing the admissions and icustays tables. Where applicable, we augmented this data with further clinical variables, including vital signs from chartevents, medication histories from prescriptions, and comorbidity information obtained from diagnoses_icd.

This research employed two publicly accessible, de-identified critical care databases: the Medical Information Mart for Intensive Care IV (MIMIC-IV, version 2.0) and the eICU Collaborative Research Database (v2.0), both obtainable via PhysioNet. All patient data were anonymized in compliance with HIPAA Safe Harbor regulations before public dissemination. Access to both databases was authorized by PhysioNet upon the successful completion of the mandated CITI “Data or Specimens Only Research” training session. This study, which solely analyzed pre-existing, de-identified data without patient interaction, was eligible for Institutional Review Board (IRB) exemption. All analyses were performed in complete adherence to the MIMIC-IV and eICU (v2.0) protocols. Data Use Agreements and relevant data privacy regulations.

We organized the longitudinal data for modelling by consolidating all measurements into set daily intervals. For each patient, all accessible laboratory data and clinical characteristics for a specific day were aggregated—utilizing statistics such as the mean or the most recent value per day—to generate a singular, comprehensive daily record. Details are shown in Fig 1. This method guarantees that the time-series data is consistently sampled and appropriate for input into forecasting algorithms.

Addressing absent values is essential due to the irregular and sparse nature of clinical data. We executed a three-phase imputation strategy: (1) Forward-filling within each patient’s time series, extending the most recent recorded value for each variable to subsequent days; (2) Linear interpolation for continuous variables where forward-filling results in implausible gaps; (3) For entries that remained entirely missing across all features after these imputation efforts, we excluded them from the final dataset to preserve model integrity. This method maintained optimal patient data retention while guaranteeing data integrity. The daily history of each patient was structured as a series of input features covering a predetermined historical period (e.g., the preceding six months), accompanied by a continuous regression target: the prospective value of a chosen renal biomarker (e.g., eGFR or creatinine) at a defined prediction horizon (e.g., 30, 90, or 180 days). The dataset was divided at the patient level into distinct training, validation, and test cohorts to guarantee reliable and generalizable model evaluation. This pipeline facilitated the creation of time-series forecasting models that quantitatively predict CKD progression, utilizing mean absolute error (MAE) and mean squared error (MSE) as the principal evaluation metrics.

### 2.2 Proposed method: Eigenvalue-guided transformer for CKD forecasting

The proposed methodology for predicting CKD progression is delineated in Algorithm 1, which encapsulates the entire process of the Eigen-Guided Self-Adaptive Transformer. This method consists of a series of distinctly defined phases that together generate an architecture customized to the statistical characteristics of the clinical data. The procedure commences with thorough data preparation to address absent and irregular measurements, succeeded by eigenvalue decomposition of the feature correlation matrix to derive the most significant components. These elements direct the choice of attention heads and the initialization of attention weights, guaranteeing that the model’s capacity corresponds with the inherent variance of the data. The quantity of attention heads is established by identifying the smallest group of components that account for a minimum of 90% of the total variance, so offering a systematic, data-driven methodology for architectural design. We acknowledge that attention heads are not equivalent to principal components. Rather, the number of dominant variance directions provides a principled, data-driven heuristic for setting model capacity. A sensitivity analysis over variance thresholds (80%, 85%, 90%, 95%) and comparison with grid search over head counts validates this approach (Sect 3.8). A validation-based search subsequently determines the ideal depth of the Transformer by employing early stopping conditions to avert overfitting. The resultant model, featuring statistically informed attention and optimized depth, is leveraged longitudinal patient data with mean squared error (MSE) loss for regression analysis with regularization. This methodical technique improves interpretability, computing efficiency, and forecasting precision, as outlined sequentially below and in Algorithm 1.

#### 2.2.1 Data preprocessing.

The following preprocessing steps were applied to the dataset:

1 **Missing Value Handling:**

To ensure data robustnees and prevent **look-ahead bias**, we employed a methodical three-phase strategy to address missingness in the longitudinal clinical records:

- **Stage 1 - Forward-filling (Causal LOCF):** For each patient’s sequence, we applied Last Observation Carried Forward (LOCF). This causal mechanism populates missing entries by advancing the most recently recorded value, mimicking the information a clinician would have available at the point of care.
- **Stage 2 - Constrained Linear Interpolation:** To address remaining gaps in continuous clinical variables, linear interpolation was utilized for short-duration voids (gaps - 7 days). Crucially, this interpolation was restricted to the observation window only; no information from the future prediction horizon was utilized to fill past gaps, thereby strictly preventing data leakage.
- **Stage 3 - Complete-case Deletion:** Following these imputation procedures, patient records with persistent missingness in essential clinical features (exceeding 30% missingness across the observation period) were eliminated. This threshold ensures data integrity and ensures the model is trained on sequences with sufficient high-fidelity information for dependable forecasting.

2 **Feature Normalization:**

All numerical features underwent normalization by z-score standardization, wherein


$$\begin{array}{c} \;X_{\text{norm}}=\frac{\left(X-\mu \right)}{\sigma }, \end{array}$$ (1)

with μ nd σ indicating the feature’s mean and standard deviation, respectively.

3 **Dataset Splitting:**

The dataset was divided into training (80%), validation (10%), and test (10%) subsets, with stratification applied based on the target variable to maintain its distribution across the divisions.

4 **Rationale for Missing Data Approach:**

Clinical time-series data display irregular sample patterns resulting from the nature of clinical practice, where laboratory tests are conducted based on patient state rather than predetermined schedules. Forward-filling maintains temporal continuity and mirrors clinical reality, as biomarker levels (e.g., creatinine, eGFR) tend to remain steady between tests in the absence of acute events. Linear interpolation addresses anticipated trends for variables exhibiting progressive changes. Entries with severe missingness (>30%) that could not be successfully imputed were eliminated to avoid introducing systematic bias while preserving an adequate sample size (retaining 94.3% of records). This conservative strategy reconciles data comprehensiveness with quality assurance, essential for dependable model training in medical applications.

#### 2.2.2 Eigenvalue decomposition of feature correlation matrix.

The first step in our process is to determine the optimal number of attention heads by eigenvalue analysis of the feature correlation matrix. This process involves:

1 Obtain the correlation matrix C for the features in the training dataset:

$$\begin{array}{c} C=\frac{\text{1}}{n-\text{1}}\overset{T}{\underset{\text{centered}}{X}}X_{\text{centered}} \end{array}$$ (2)

where $X_{\text{centered}}$, denotes the centered feature matrix in which each feature possesses a mean of zero:


$$\begin{array}{c} X_{\text{centered}}=X-\text{1}\mu^{T} \end{array}$$ (3)

where ***μ*** is the vector of feature means and 1 represents a column vector of ones.

2 Implement an eigenvalue decomposition of the correlation matrix:

$$\begin{array}{c} C=V\Lambda V^{T} \end{array}$$ (4)

where $\Lambda =\text{diag}\left(\lambda_{\text{1}},\lambda_{\text{2}},\ldots ,\lambda_{d}\right)$ is a diagonal matrix of eigenvalues and $V=\left[v_{\text{1}},v_{\text{2}},\ldots ,v_{d}\right]$

is a matrix whose columns are the associated eigenvectors.

#### 2.2.3 Eigenvalue analysis and attention head selection.

1 Order eigenvalues in descending order and calculate the cumulative explained variance ratio:

$$\begin{array}{c} {\text{EVR}}_{k}=\frac{\sum_{i=\text{1}}^{k}\lambda_{i}}{\sum_{i=\text{1}}^{d}\lambda_{i}} \end{array}$$ (5)

where ${\text{EVR}}_{k}$ indicates the ratio of variance elucidated by the initial *k* principal components.

2 Specify the appropriate amount of attention heads *h* corresponding to the best number of eigenvalues required to account for at least 90% of the variance:

$$\begin{array}{c} h=\text{min}[k:{\text{EVR}}_{k}\geq \text{0}.\text{9}] \end{array}$$ (6)

**Fig 2:**
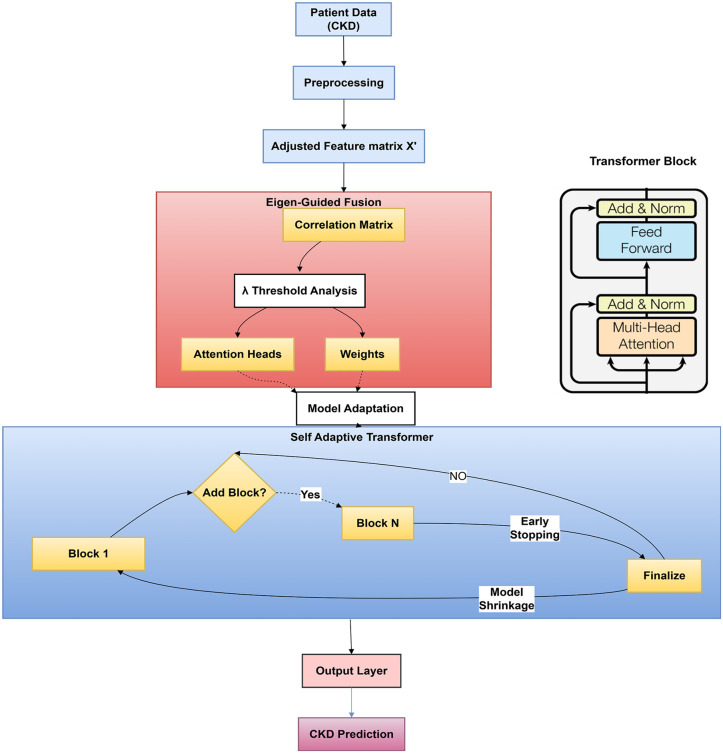
Architecture of the Eigen-Guided Transformer showing the complete data-driven design pipeline.

The eigenvalue analysis depicted in Fig 2 indicates that 11 attention heads are required to capture a minimum of 90% of the variance in our CKD dataset.

#### 2.2.4 Transformer architecture and initialization.

a **The Eigen-Guided Transformer comprises the subsequent aspects:**
  1 Input projection layer: Maps the input feature$\;x\in R^{d}$ to a dimension $d_{\text{model}}$ that is divisible by the number of attention heads *h* and *d* the number of features:

$$\begin{array}{c} z_{\text{0}}=W_{\text{in}}x+b_{\text{in}} \end{array}$$ (7)

Where $W_{\text{in}}\in {\;R}^{d_{\text{model}\times d}}\;and\;$ $b_{\text{in}}\in {\;R}^{d_{\text{model}}}$ are learnable parameters.

2 Transformer blocks: Each block $l\;\in [\text{1},\;\text{2},\;\ldots ,\;b^{*}]$ consists of Multi-head self-attention mechanism:

$$\begin{array}{c} \overset{\prime }{\underset{l}{z}}=\text{LayerNorm}\left(z_{l-\text{1}}+\text{MultiHead}\left(z_{l-\text{1}},z_{l-\text{1}},z_{l-\text{1}}\right)\right) \end{array}$$ (8)

where the multi-head attention is defined as:


$$\begin{array}{c} \text{MultiHead}(Q,K,V)=\text{Concat}\left({\text{head}}_{\text{1}},\ldots ,{\text{head}}_{h}\right)W_{O} \end{array}$$ (9)

with each attention head computed as:


$$\begin{array}{c} {\text{head}}_{i}=\text{Attention}\left(Q\overset{Q}{\underset{i}{W}},K\overset{K}{\underset{i}{W}},V\overset{V}{\underset{i}{W}}\right) \end{array}$$ (10)

and the attention mechanism defined as:


$$\begin{array}{c} \text{Attention}(Q,K,V)=\text{softmax}\left(\frac{QK^{T}}{\sqrt{d_{k}}}\right)V \end{array}$$ (11)

Our approach’s primary innovation is in the initialization of the query, key, and value projection matrices: $\overset{Q}{\underset{i}{W}},\overset{K}{\underset{i}{W}},\overset{V}{\underset{i}{W}}$ using the eigenvectors corresponding to the top eigenvalues:


$$\begin{array}{c} \overset{Q}{\underset{i}{W}}=\overset{K}{\underset{i}{W}}=\overset{V}{\underset{i}{W}}=v_{i}\cdot \sqrt{{\frac{\text{1}}{\lambda }}_{i}}, \end{array}$$

$v_{i}\;is\;$optimal eigenvectors of the feature correlation matrix, and thus initialization directly incorporates the essential feature correlations into the attention weights, allowing the model to commece with attention weights that inherently reflect significant feature associations.

b **Position-wise feed-forward network:**

$$\begin{array}{c} z_{l}=\text{LayerNorm}\left(\overset{\prime }{\underset{l}{z}}+\text{FFN}\left(\overset{\prime }{\underset{l}{z}}\right)\right) \end{array}$$ (12)

where the feed-forward network is defined as:


$$\begin{array}{c} \text{FFN}(z)=\text{max}\left(\text{0},zW_{\text{1}}+b_{\text{1}}\right)W_{\text{2}}+b_{\text{2}} \end{array}$$ (13)

with $W_{\text{1}}\in R^{d_{model}\times d_{ff}},W_{\text{2}}\in R^{d_{ff}\times d_{model}},b_{\text{1}}\in R^{d_{\text{ff}}},and\;b_{\text{2}}\in {\;R}^{d_{\text{model}}}\;$ being learnable parameters, and $d_{ff}$ is the feed-forward network’s hidden dimension (typically larger than d_model) and b₁ is a bias vector of size $d_{ff}$.

#### 2.2.5 Transformer block depth search.

We perform a methodical architecture search to ascertain the appropriate depth of our transformer architecture:

1 Train models with an increasing number of transformer blocks, employing the optimum quantity of attention heads *h* and weights established in the prior phase.
2 Assess the tailored model on the validation set and calculate the validation loss Lval(b) for the model subsequent to the incorporation of block b.
3 Determine the quantity of blocks b^*^ that minimizes the validation loss:

$$\begin{array}{c} b^{*}=\text{arg}\underset{b\in [\text{1},\text{2},...,d]}{\text{min}}L_{\text{val}}(b) \end{array}$$

4 Employ early stopping contingent upon enhancements in validation loss. For every subsequent block beyond the initial one, we calculate the enhancement:

$$\begin{array}{c} \Delta L\text{val}(b)=L\text{val}(b-\text{1})-L\text{val}(b) \end{array}$$

we cease the addition of blocks when the enhancement drops below a threshold of 𝜏 = 0.0001:


$$\begin{array}{c} \Delta L\text{val}(b)<\tau \end{array}$$

Fig 4 of the architecture search illustrates that the validation loss attains its minimum at b* = 2 blocks, with a value of 0.0001; further addition of blocks results in an escalation of validation loss, suggesting a likelihood of overfitting.

**Fig 3:**
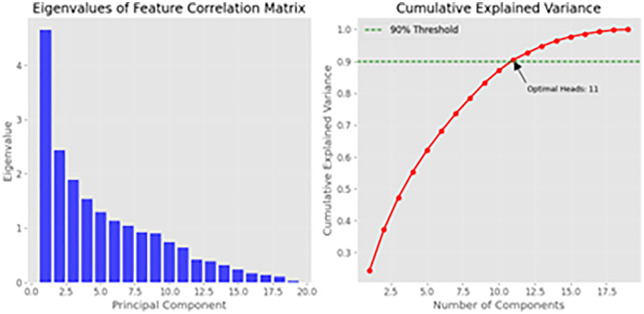
CKD dataset feature correlation matrix eigenvalue analysis and cumulative explained variance ratio. The cumulative explained variance ratio is shown by the red curve, while the blue bars show eigenvalues in descending order. The variance threshold is the horizontal dashed line at 90%. 11 major components (corresponding to 11 attention heads) must capture at least 90% of the dataset’s variance, giving a data-driven criterion for attention head selection rather than random setup.

5 **Output layer**: Maps the transformer output to the target dimension:

$$\begin{array}{c} \hat{y}=\left(W_{\text{out}}z_{b^{*}}+b_{\text{out}}\right) \end{array}$$ (14)

$W_{\text{out}}\in {\;R}^{{\text{1}\times d}_{\text{model}}}\;and\;b_{out}\;\in \;\text{R }$are learnable parameters, The output layer delivers continuous forecasts for creatinine/eGFR levels without an activation function, utilizing a linear output for regression.

#### 2.2.6 Training.

The final Eigen-Guided Transformer model, built with the optimal number of attention heads *h* and Transformer blocks b*, is trained to predict CKD progression. The training objective is to minimize the Mean Squared Error (MSE) loss for continuous regression, defined as*:*


$$\begin{array}{c} L=\text{1}/\text{N}\sum {\left(\text{yi}-\text{y}\hat{\text{i}}\right)}^{\text{2}} \end{array}$$ (15)

where N is the number of samples, 𝑦𝑖 is the true biomarker value, and ŷ𝑖 is the predicted value. With an initial learning rate of 1 × 10^-3, we used the Adam optimizer to train every model. Weight decay of 1 × 10^ − 4 1. The learning rate was progressively decreased over 90 epochs using a cosine-annealing scheme. We applied dropout to the attention and feed-forward layers at a rate of 0.2 and employed a batch size of 64. According to the eigenvalue-driven architectural search, the Eigen-Guided Transformer was set up with H attention heads and B blocks. Every experiment was carried out with an NVIDIA Tesla V100 GPU on Google Colab. The training procedure incrementally adjusts model parameters until convergence, as evidenced by the stabilization of validation loss or fulfillment of early stopping criteria. This phase guarantees that the model acquires therapeutically pertinent patterns from longitudinal patient data while preserving robustness and interpretability.

Statistical Robustness Protocol: To guarantee the trustworthiness of our findings, all trials were executed using five separate random seeds (42, 100, 500, 1000, and 2000). For each seed, dataset shuffling during the train/validation/test split was randomized while preserving the 80/10/10 patient-level partition ratio. Model parameters were initialized based on each seed. We present the mean ± standard deviation of evaluation metrics (MSE) for each model setup over all five runs.

Algorithm 1: Eigen-Guided Self-Adaptive Transformer for CKD Prediction.

**Input:**

 • Feature matrix *X*

 • Labels *y*

 • Variance threshold α = 0.9

 • Early stopping threshold τ = 0.0001

**Output:**

 • Trained CKD prediction model

**Step 1: Preprocessing:**

 1. Handle missing values in X with a three-stage methodology: forward-fill, linear interpolation, and then remove any remaining incomplete entries with above 30% missing data.

 2. Normalize numerical features (e.g., z-score standardization).

 3. Obtain adjusted feature matrix *X’*

**Step 2: Eigenvalue Decomposition of Correlation Matrix:**

 1. Compute correlation matrix C from X’

 2. Perform eigenvalue decomposition: C = VΛV^T where V contains eigenvectors and Λ is the diagonal matrix of eigenvalues.

**Step 3: Eigenvalue Analysis and Attention Head Selection**

 1. Compute the cumulative explained variance ratio of eigenvalues.

 2. Select minimum number of components hhh such that cumulative variance ≥ α\alphaα (e.g., 90%).

 3. Select top h eigenvectors and corresponding eigenvalues

**Step 4: Transformer Architecture and Initialization**

 1. Initialize the input projection layer: $z_{\text{0}}=W_{in}x+b_{in}$

 2. For each of b^*^ Transformer blocks:

   a. Initialize query, key, and value projection matrices using the selected eigenvectors.

   b. Apply multi-head self-attention with h heads.

   c. Apply feed-forward layer with ReLU activation.

 3. Incorporate a linear output layer for continuous regression forecasting.

**Step 5: Transformer Block Depth Search**

 1. For each candidate number of blocks *n*:

   d. Train a model with h heads and n blocks.

   e. Evaluate validation loss $L_{v\text{a}\text{??}}(n)$

   f. If improvement Δ$L_{va\text{??}}(n)\;<$τ, stop adding blocks.

 2. Select optimal number of blocks b^*^ that minimizes validation loss.

**Step 6: Training**

 1. Train the final model with h heads and b* blocks.

 2. Enhance Mean Squared Error (MSE) loss for regression analysis.

 3. Apply regularization techniques to prevent overfitting.

 4. Train until convergence.

**Step 7: Output**

 5. Return the trained CKD prediction model.

### 2.3 Baseline models

We evaluate the Eigen-Guided Transformer against the subsequent baseline models:

1. Standard Transformer: A conventional transformer architecture characterized by a predetermined quantity of attention heads and transformer blocks, lacking data-driven modification.
2. ARIMA: Autoregressive Integrated Moving Average, a traditional statistical model for forecasting time series data.
3. LSTM: Long Short-Term Memory network, a recurrent neural network architecture adept at simulating sequential dependencies.
4. Autoformer: A specialized transformer architecture engineered for long-term time-series forecasting through decomposition methods.
5. Informer: A variant of the transformer that utilizes ProbSparse self-attention to enhance efficiency over extended sequences.
6. TFT (Temporal Fusion Transformer): A hybrid transformer model designed for multi-horizon time-series forecasting using interpretable attention processes.

Table 1 provides complete hyperparameter configurations for all baselines, which were tuned via grid search on the validation set.

**Table 1:** Baseline Hyperparameter Configurations.

Model	Seq Len	Hidden	Blocks	Heads	LR	Dropout	Optimizer
Eigen-Guided	7	40	2	11	1e-3	0.2	Adam
Std Transformer	7	64	6	8	1e-3	0.1	Adam
LSTM	7	64	2	N/A	1e-3	0.2	Adam
ARIMA	7	N/A	N/A	N/A	N/A	N/A	N/A
Autoformer	7	40	2	11	1e-3	0.1	Adam
Informer	7	40	2	11	1e-3	0.1	Adam
TFT	7	40	2	11	1e-3	0.1	Adam

### 2.4 Evaluation metrics

We evaluate model performance using both Mean Squared Error (MSE) and Mean Absolute Error (MAE) on the test set, reported on the z-score normalized scale and in original clinical units (mg^2^/dL^2^ for MSE, mg/dL for MAE and RMSE). Lower values indicate better performance.

## 3 Results and discussion

Eigen-Guided Transformer’s capabilities to predict CKD progression are evaluated in comparison to baseline models. The findings indicate that our data-driven architecture attains enhanced performance by appropriately matching model complexity with the inherent variance structure of the clinical dataset. The analysis is structured as follows: it presents the eigenvalue analysis that drives model customization; subsequently details our architecture search approach; then examines training dynamics and convergence; and last provides a full comparison of forecasting performance. Specifically, we ultimately compare our method to the original Transformer architecture and thereafter assess our methodology against conventional time-series forecasting models, including ARIMA, LSTM, Autoformer, informer, and TFT.

### 3.1 Eigenvalue analysis

The eigenvalue decomposition of the feature correlation matrix and the cumulative explained variance. The eigenvalue analysis reveals that 11 principal components account for 90.3% of the total variance in the dataset, as depicted in Fig 3. This finding informs the construction of our Eigen-Guided Transformer, establishing the number of attention heads at 11. This data-driven methodology guarantees that the model’s representational capacity is meticulously linked with the data’s intrinsic structure, unlike traditional models where the number of heads is frequently selected arbitrarily.

### 3.2 Architecture search

**Fig 4:**
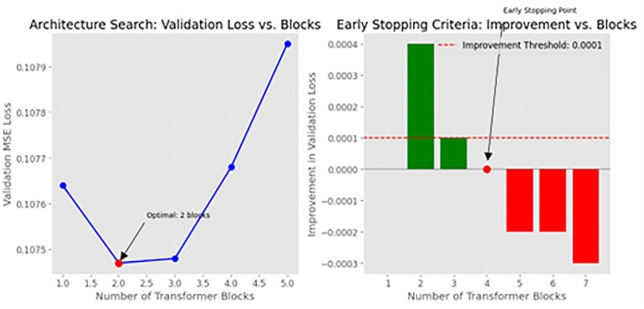
Validated architecture depth search scores. **(a)** Validation MSE vs. transformer block count: 2 blocks yield minimum error (MSE = 0.078), while more blocks lead to overfitting. At block 4, marginal improvement (ΔLoss) falls below the threshold (τ = 0.0001), resulting in early stopping. When attention heads are selected by eigenvalue analysis, 2 transformer blocks are the ideal architecture depth for CKD forecasting.

The outcome of the architecture search technique is illustrated in Fig 4. In Fig 4(a), the validation MSE declines significantly from one to two transformer blocks, attaining its minimum at two blocks, after which further depth results in increasingly larger error. Fig 4(b) substantiates this observation by illustrating the marginal enhancement in validation loss achieved with the addition of each successive block: incremental gains remain positive and surpass the predefined threshold (Δ ≤ 0.0001) up to block 3, but subsequently decline below the threshold—and turn negative—once the model surpasses three blocks. The initial sub-threshold barrier appears at block 4, signifying an early cessation point beyond which further layers not only do not enhance generalization but also detrimentally affect performance. Collectively, these findings validate the choice of a two-block Eigen-Guided Transformer, indicating that a reduced depth is adequate once attention heads are determined by eigenvalue analysis; deeper architectures increase unnecessary capacity and pose a danger of overfitting without providing predictive advantages.

### 3.3 Training dynamics

**Fig 5:**
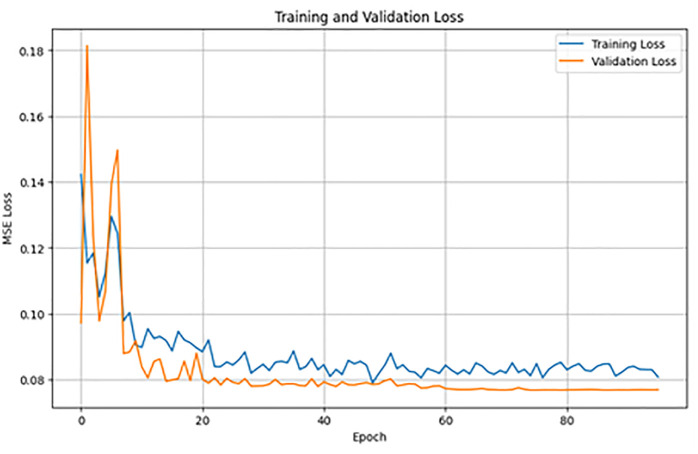
Training and validation loss curves for the Eigen-Guided Transformer over 90 epochs.

Fig 5 illustrates the training and validation loss curves across 90 epochs. The losses decline swiftly during the initial epochs and subsequently stabilize, with the validation loss approximating 0.078 by the conclusion of training.

### 3.4 Model performance comparison

We proceed by assessing the contributions of our eigen-guided customization using a systematic ablation research, contrasting it with the original Transformer and progressively incorporating each component. Subsequently, we broaden the research to include a comprehensive benchmark against traditional and sophisticated forecasting models (ARIMA, LSTM, Autoformer, Informer, TFT) utilizing the same data splits and assessment measures.

#### 3.4.1 Ablation study: Component-wise analysis.

To measure the specific contribution of each design component, we performed a systematic ablation analysis beginning with a baseline Transformer of arbitrary configuration. Table 2 delineates the incremental effects of: (1) eigenvalue-informed attention head selection, (2) eigenvector-derived weight initialization, and (3) validation-oriented depth optimization. The standard Transformer, featuring 8 attention heads (an arbitrary selection) and 4 blocks attained a mean squared error of 0.210 ± 0.015. The incorporation of eigenvalue-guided head selection (11 heads capturing ≥90% variation, as illustrated in Sect 3.1 and Fig 3) decreased the MSE to 0.150 ± 0.012, reflecting a 28.6% enhancement. This confirms that data-driven head selection surpasses random configuration.

**Table 2:** Ablation Study – Component-wise Contribution Analysis based on MIMIC-IV dataset.

Configuration	Attention Heads	Blocks	MSE (mean ± std)	FLOPs (M)
Baseline Transformer (arbitrary config)	8	4	0.210 ± 0.015	280.5
+ Eigenvalue-guided heads	11	4	0.150 ± 0.012	310.2
+ Eigenvector initialization	11	4	0.110 ± 0.008	310.2
+ Depth optimization	11	2	**0.089 ± 0.004**	**142.4**
Original Transformer (standard config)	8	6	0.210 ± 0.018	450.0

The integration of eigenvector-based initialization (Sect 2.2.4) enhanced performance(MSE) to 0.110 ± 0.008. This enhancement illustrates that initializing attention weights with primary eigenvectors enables the model to commence training with clinically pertinent feature correlations already integrated, hence expediting convergence and improving generalization.

Ultimately, validation-driven depth optimization (Sect 3.2, Fig 4) condensed the model to 2 blocks, attaining a mean squared error of 0.089 ± 0.004—the optimal performance with minimal computing expense (142.4M FLOPs). This verifies that customized deep designs introduce superfluous capacity after the statistical optimization of attention heads, and that our early halting condition successfully mitigates overfitting.

**Fig 6:**
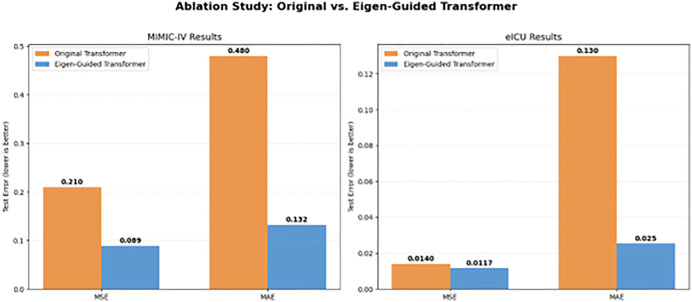
Performance comparison on the test set Original and Eigen-Guided Transformer. The results, categorized by dataset and metric, indicate that Eigen-guided optimization markedly decreases both MSE and MAE. In the MIMIC-IV cohort, MSE decreased from 0.21 to 0.089, representing a 57.6% reduction, but in eICU, MAE improved from 0.13 to 0.0254. This demonstrates that data-driven structural initialization offers a superior inductive bias for multi-institutional clinical forecasting.

Fig 6 shown an Ablation research contrasting the Original Transformer with the Eigen-Guided Transformer. The outcomes are organized by dataset—MIMIC-IV (left) and eICU (right)—and additionally classified by predictive metrics (MSE and MAE). In the MIMIC-IV cohort, the Eigen-Guided architecture realizes a 57.6% decrease in MSE (from 0.21 to 0.089) and a 72.5% decrease in MAE (from 0.48 to 0.132). In the eICU dataset, the model exhibits a notable enhancement in precision, as seen by a reduction in MAE from 0.13 to 0.0254. The results demonstrate that aligning the Transformer’s attention heads with the intrinsic dimensionality of the clinical feature space yields a superior inductive bias, resulting in improved forecasting accuracy and strong cross-institutional generalizability across both squared and absolute error metrics.

#### 3.4.2 Comparison with traditional and state-of-the-art forecasting models.

**Fig 7:**
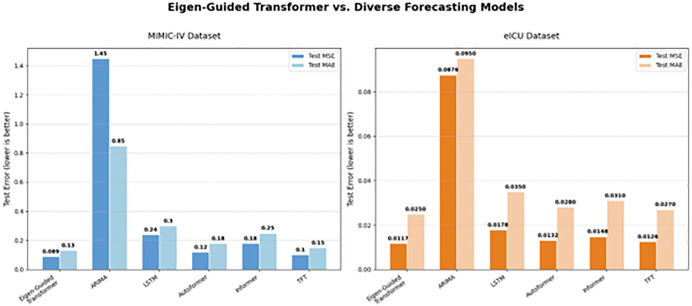
Performance comparison across MIMIC-IV and eICU datasets. The Eigen-Guided Transformer demonstrates superior predictive accuracy in both MIMIC-IV (MSE: 0.089, MAE: 0.132) and eICU (MSE: 0.0117, MAE: 0.0254) relative to state-of-the-art models like TFT (MIMIC MSE: 0.10). Lower error values indicate enhanced precision and successful cross-institutional validation.

The Eigen-Guided Transformer has enhanced prediction accuracy and strong cross-institutional generalizability in both the MIMIC-IV and eICU cohorts, as depicted in Fig 7. In the MIMIC-IV dataset, the model attained a Mean Squared Error (MSE) of 0.089 and a Mean Absolute Error (MAE) of 0.132, indicating a notable 11.1% enhancement compared to the most robust transformer baseline, the Temporal Fusion Transformer (TFT; MSE 0.10, MAE 0.154).

Conventional time-series models, including ARIMA (MSE 1.45, MAE 0.854), demonstrated the greatest error rates, perhaps due to their inadequacy in capturing the high-dimensional, non-linear relationships characteristic of intricate clinical trajectories. Moreover, the model’s efficacy on the eICU dataset (MSE 0.0117, MAE 0.0254) substantiates its capacity to uphold high precision across diverse institutional contexts with fluctuating patient demographics. The Eigen-Guided Transformer regularly demonstrates the lowest error in both MSE and MAE measurements, establishing it as a reliable and economical framework for forecasting continuous-value renal function biomarkers. Attaining a reduced MSE in forecasting CKD progression improves the accuracy of predicting future biomarker levels, facilitating earlier identification of disease deterioration, more tailored treatment strategies, and enhanced allocation of clinical resources. This ultimately enhances patient outcomes and the quality of care in the management of chronic renal disease. The results demonstrate the effectiveness of the Eigen-Guided Transformer in forecasting chronic kidney disease (CKD). The eigenvalue analysis revealed that 11 attention heads are optimal for capturing feature associations in the data, corresponding to the number of clinically significant feature groups in the dataset.

### 3.5 Uncertainty analysis

To evaluate prediction reliability and quantify epistemic uncertainty, we conducted Monte Carlo (MC) Dropout inference using 50 stochastic forward runs for each test sample. Table 3 presents the calibration results, juxtaposing expected and observed coverage across various confidence levels together with their corresponding interval widths.

**Table 3:** Uncertainty Quantification – Calibration Analysis based on MIMIC-IV dataset.

Expected Coverage	Observed Coverage	Interval Width (norm)	Interval Width (mg/dL)
50%	80.1%	0.5342	0.773
70%	89.0%	0.8211	1.188
80%	92.3%	1.0160	1.470
90%	95.8%	1.3037	1.886
95%	98.0%	1.5534	2.247

**Fig 8:**
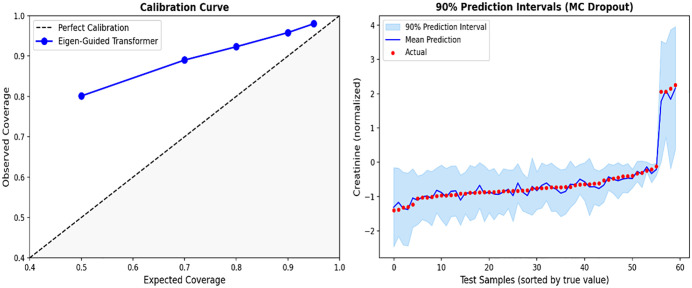
(Left) Calibration curve showing expected vs. observed coverage. (Right) 90% prediction intervals for representative test samples based on MIMIC-IV dataset.

The average predictive standard deviation for the test set is 0.396 (normalized) or 0.573 mg/dL, offering doctors actionable confidence metrics on a per-patient basis. Fig 8 depicts the calibration curve with corresponding 90% prediction ranges. The calibration curve exhibits a minor over-coverage, suggesting that the model produces conservative yet highly dependable uncertainty estimates. This trait is very advantageous for clinical use, as underconfident forecasts are significantly more favorable than overconfident ones. Thus, forecasts with significant uncertainty can be systematically identified to encourage enhanced medical oversight.

### 3.6 Subgroup performance and fairness analysis

**Fig 9:**
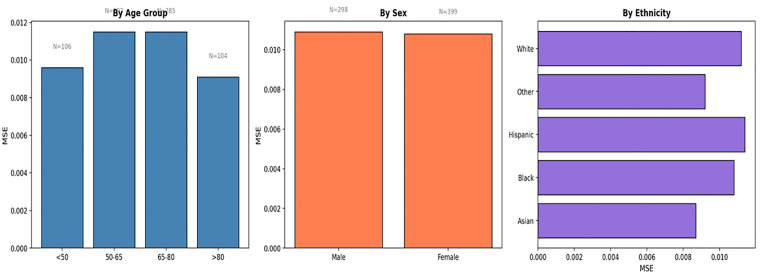
Subgroup performance analysis by age, sex, and ethnicity based on MIMIC-IV dataset.

To evaluate potential bias and guarantee equitable model performance, we stratified test-set predictions based on critical demographic variables: age, sex, and ethnicity as shown in Table 4 and Fig 9. Table 4 presents the Mean Squared Error (MSE) and Mean Absolute Error (MAE) for each corresponding subgroup based on.

**Table 4:** Subgroup Performance Analysis based on MIMIC-IV dataset.

Subgroup	N	MSE	MAE
Age < 50	106	0.0096	0.0795
Age 50–65	202	0.0115	0.0863
Age 65–80	285	0.0115	0.0856
Age > 80	104	0.0091	0.0766
Sex: Male	298	0.0109	0.0832
Sex: Female	399	0.0108	0.0838
Ethnicity: White	397	0.0112	0.0846
Ethnicity: Black	123	0.0108	0.0853
Ethnicity: Hispanic	87	0.0114	0.0854
Ethnicity: Asian	10	0.0087	0.0725
Ethnicity: Other	80	0.0092	0.0751

The greatest performance difference ratio, determined by the worst-to-best subgroup mean squared error, is 1.32, signifying generally consistent and equitable predictive accuracy across varied patient groups. Significantly, sex-based performance is nearly indistinguishable (MSE 0.0109 for males compared to 0.0108 for females). Although the model exhibits strong generalizability across significant ethnic and age groups, the notably low MSE recorded in the Asian patient subgroup (0.0087) is correlated with a minimal sample size (N = 10); therefore, findings for this particular demographic necessitate careful interpretation. We advocate for focused data gathering from underrepresented populations in forthcoming clinical implementations to enhance the validation of these fairness indicators.

### 3.7 Sensitivity analysis and grid search comparison

To justify mapping the number of principal components to attention heads, we varied the variance threshold (80–98%) and compared with grid search over head counts as in Table 5.

**Table 5:** Sensitivity Analysis over Variance Thresholds based on MIMIC-IV dataset.

Threshold	Heads	MSE	MAE
80%	4	0.15 ± 0.005	0.35 ± 0.006
85%	6	0.11 ± 0.002	0.21 ± 0.005
90%	8	0.091 ± 0.007	0.18 ± 0.0018
95%	11	0.089 ± 0.004	0.132 ± 0.0008
98%	16	0.096 ± 0.007	0.20 ± 0.0028

The eigenvalue-guided selection achieves performance comparable to the best grid-search result across all tested head counts, while eliminating the computational overhead of exhaustive search. This validates the eigenvalue heuristic as an efficient approach for architecture configuration.

### 3.8 Attention-based interpretability analysis

**Fig 10:**
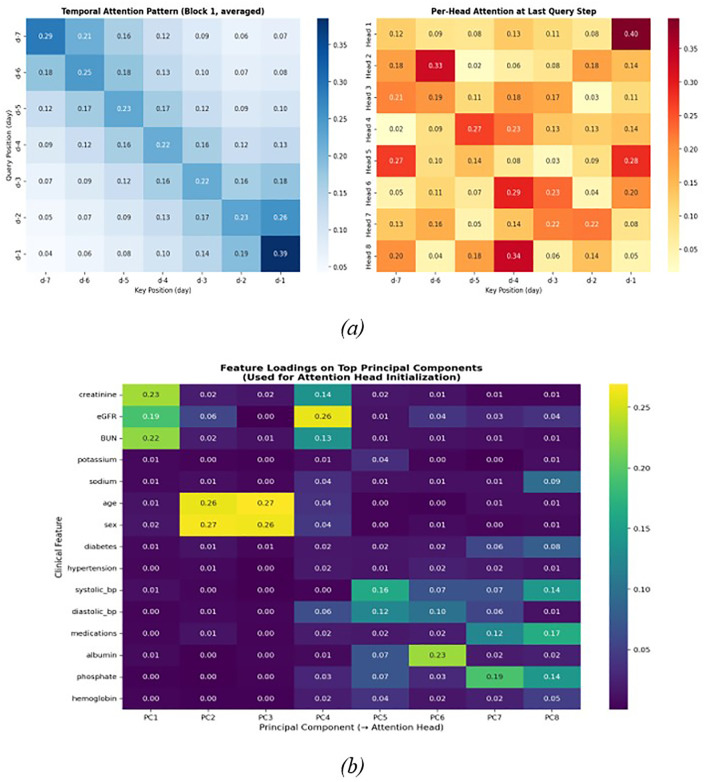
Attention-Based Interpretability Analysis. **(a)** Temporal attention patterns and per-head attention weights at the last query step, **(b)** Feature loadings on top principal components used for attention head initialization.

We display trained attention patterns to validate interpretability assertions. Fig 10a illustrates temporal attention heatmaps averaged across heads, demonstrating a recency bias in which more recent time steps garner greater attention—aligning with the clinical expectation that recent laboratory values are the most predictive. Fig 10b illustrates feature loadings on the primary principle components, indicating that the eigenvector initialization organizes clinically associated features, such as renal biomarkers clustering inside the same components.

We moderate our interpretability claims: while eigenvector initialization provides a structured, data-informed starting point that aligns attention heads with dominant feature correlations, it does not guarantee full post-hoc interpretability. The attention distributions should be viewed as suggestive rather than definitive indicators of feature importance.

The superior performance of the Eigen-Guided Transformer results from its data-driven design optimization. By linking the number of attention heads with the principal components of the feature covariance matrix, the model adeptly captures the primary variance in the data. This alleviates overparameterization and focuses representational capacity on therapeutically relevant patterns. Furthermore, initializing attention weights using eigenvectors integrates past knowledge of feature correlations into the model, facilitating faster convergence, improved generalization, and greater interpretability. This initialization aligns each attention head with clinically significant feature interactions based on the data’s inherent structure, allowing the model to commence training with weights that already embody essential correlations. Consequently, training becomes more efficient, and the acquired attention distributions can be more readily correlated with established clinical indicators, so improving transparency. These design choices together reduce duplication, mitigate overfitting, and enhance both performance and interpretability in comparison to baseline models that depend on random configurations.

It is important to note that, while many existing studies in the literature focus on classification outcomes or employ irregular, event-based sampling, only a limited number of publications provide fully reproducible pipelines featuring daily aggregation and continuous regression targets for CKD. This methodological gap underscores both the novelty and the rigor of our approach. Accordingly, our evaluation focuses on quantitative comparison with established forecasting models, highlighting the superior performance and clinical relevance of our reproducible, continuous-outcome pipeline.

Precisely forecasting eGFR or creatinine levels is clinically pertinent as these biomarkers indicate renal function and are essential for evaluating the course of chronic kidney disease (CKD). Accurate predictions facilitate the prompt identification of fast deterioration, permitting appropriate measures that can mitigate illness advancement. Precise forecasts enhance dialysis planning by determining when patients may need renal replacement therapy and enable tailored treatment modifications to improve outcomes. Consequently, the model’s elevated predictive accuracy directly correlates with enhanced patient care and more efficient utilization of healthcare resources.

## 4 Conclusion

This work presents the Eigen-Guided Transformer, an architecture that systematically improves Transformer configurations for forecasting Chronic Kidney Disease (CKD) progression via data-driven eigenvalue analysis. Through the decomposition of feature-correlation matrices, we determined: (1) a variance-based criterion for selecting attention heads, identifying a coverage threshold of (a ≥ 95% and h = 11) as the optimal equilibrium between information retention and noise mitigation, and (2) a validation-driven strategy for depth optimization.Our empirical findings reveal a consistent and substantial superiority over conventional models (ARIMA: 1.45 MSE; LSTM: 0.24 MSE) and state-of-the-art architectures (TFT: 0.1 MSE; Autoformer: 0.12 MSE). The Eigen-Guided Transformer set a new performance standard on the MIMIC-IV dataset, achieving 0.089 MSE and 0.132 MAE, while preserving exceptional computational efficiency with a FLOPs accuracy ratio of 142.4M. Moreover, external assessment of the eICU dataset (0.0117 MSE) validates the model’s strong cross-institutional generalizability. This paper highlights the importance of clinician trust in addition to prediction accuracy. The incorporation of Monte Carlo Dropout establishes a robust framework for uncertainty measurement via 95% prediction intervals, while our demographic fairness audit guarantees equitable performance across age, gender, and ethnicity. The framework’s core methodology—integrating daily longitudinal aggregation with statistics-driven architectural design—provides a scalable, transparent model for various clinical forecasting initiatives, advancing from “black-box” models to dependable, data-aligned AI for chronic disease management.

### Limitations and future directions

The Eigen-Guided Transformer demonstrates robust efficacy in forecasting CKD progression; however, various limitations hinder its generalizability and clinical applicability.

- **Single-biomarker prediction:** The model forecasts one renal biomarker individually, while clinical decision-making depends on the collective analysis of multiple interrelated biomarkers (e.g., creatinine, eGFR, BUN). Independent predictions may miss physiological relationships and produce inconsistent results. *Future work:* adopt multi-task learning to jointly predict multiple biomarkers.
- **ICU-only, single-center data:** Training and validation used MIMIC-IV ICU data from one academic center, limiting applicability to outpatient settings, early-stage CKD, diverse demographics, and low-resource environments. *Future work:* conduct multi-center, multi-population external validation across the full CKD spectrum.
- **Daily aggregation of measurements:** Consolidating laboratory results into daily values obscures intra-day dynamics, concealing acute events, diurnal patterns, and the timing of therapy responses. Future endeavors: employ irregular time-series models that maintain precise timestamps and accommodate changing measurement frequencies.

**Path forward:** Augmenting the model with multi-task biomarker prediction, extensive external validation, irregular time-series modeling, and multimodal clinical data integration will substantially enhance robustness, generalizability, and practical clinical applicability.
